# Are non-starters accumulating enough load compared with starters? Examining load, wellness, and training/match ratios of a European professional soccer team

**DOI:** 10.1186/s13102-023-00743-y

**Published:** 2023-10-10

**Authors:** Rafael Oliveira, Rui Canário-Lemos, Ryland Morgans, Tiago Rafael-Moreira, José Vilaça-Alves, João Paulo Brito

**Affiliations:** 1 Sports Science School of Rio Maior–Polytechnic Institute of Santarém, Rio Maior, 2040-413 Portugal; 2 Life Quality Research Centre, Rio Maior, 2040-413 Portugal; 3 The Research Centre in Sports Sciences, Health Sciences and Human Development (CIDESD), Vila Real, 5001-801 Portugal; 4 University of Trás-Os-Montes E Alto Douro, Vila Real, 5001-801 Portugal; 5 Research Group in Strength Training and Fitness Activities, GEETFAA, 5001-801 Vila Real, Portugal; 6 Football Performance Hub, University of Central Lancashire, Preston, PR1 2HE UK; 7 Research Center of the Polytechnic Institute of Maia (N2i), Maia Polytechnic Institute (IPMAIA), Castêlo da Maia, Maia, 4475-690 Portugal

**Keywords:** Fatigue, Football, Mood, Muscle soreness, Load, Sleep, Stress, Wellbeing, Training load, Load quantification, External load, Internal load, Sports training

## Abstract

**Background:**

The aims of the study were to: (i) compare accumulated load and wellness between starters and non-starters of a European professional soccer team; (ii) analyze the relationships between wellness and load measures and; (iii) compare training/match ratio (TMr) of external and internal load between starters and non-starters.

**Methods:**

Ten players were considered starters while seven were classified as non-starters over a 16-week period in which six training sessions and match day (MD) were considered in each weekly micro-cycle. The following measures were used: wellness (fatigue, quality of sleep, muscle soreness, stress, and mood); load (rated of perceived exertion (RPE), session-RPE (s-RPE), high-speed running (HSR), sprinting, accelerations (ACC) and decelerations (DEC)). Accumulated wellness/load were calculated by summing all training and match sessions, while TMr was calculated by dividing accumulated training load by match data for all load measures and each player. Mann–Whitney U test was used for wellness variables, while independent T-test was used for the remaining variables to compare groups. Moreover, relationships among variables were explored using the Spearman’s Rho correlation coefficient.

**Results:**

The main results showed that non-starters presented higher significant values for fatigue (*p* < 0.019; g = 0.24) and lower significant values for duration (*p* < 0.006; ES = 1.81) and s-RPE (*p* < 0.001; ES = 2.69) when compared to starters. Moreover, positive and very large correlation was found between quality of sleep and RPE, while negative and very large correlation were found between stress and deceleration, and mood and deceleration (all, *p* < 0.05). Finally, non-starters presented higher values in all TMr than starters, namely, RPE (*p* = 0.001; g = 1.96), s-RPE (*p* = 0.002; g = 1.77), HSR (*p* = 0.001; g = 2.02), sprinting (*p* = 0.002; g = 4.23), accelerations (*p* = 0.001; g = 2.72), decelerations (*p* < 0.001; g = 3.44), and duration (*p* = 0.003; g = 2.27).

**Conclusions:**

In conclusion, this study showed that non-starters produced higher TMr in all examined variables despite the lower match and training durations when compared with starters, suggesting that physical load was adjusted appropriately. Additionally, higher RPE was associated with improved sleep while higher number of decelerations were associated with decreased wellness, namely, stress and mood for non-starters.

## Introduction

The quantification of training and match load/demands on soccer players is a common practice [1–3]. Specifically, the monitorisation of athletes include the quantification of training/match demands (e.g., locomotor/mechanical and psychophysiological), and the wellness and readiness of players [4]. On the one hand, locomotor/mechanical demands or just physical demands are associated with external load monitoring using global positioning system (GPS) variables (e.g., distances covered at various running speeds or accelerations). While psychophysiological demands are associated with internal load/intensity monitoring employing subjective and/or objective measures (e.g. rating of perceived exertion (RPE) and heart rate, respectively) [2, 5]. While, wellness is usually measured by questionnaires as previously proposed by Hooper and Mackinnon [6] and McLean et al. [7]. These questionnaires assess various items such as fatigue, quality of sleep, muscle soreness, mood and stress [7], that may vary depending on the specific load applied [4]. Thus, internal load can be estimated from questionnaires whose initial validity presented some concerns, although these issues have recently been resolved due to numerous scientific studies demonstrating individual response sensitivity to training-load changes [8–10].

Despite the relevance that has been attributed to monitoring measures, external load monitoring often raises questions surrounding technology reliability and validity, as well as inconsistencies in those reported measures [11, 12]. However, internal load monitoring has made assumptions regarding player adherence, as well as uncertainty surrounding the impact of these measures on player performance [13]. Furthermore, despite questions over the reliability of GPS data [11] and on internal load monitoring instruments, research has supported their implementation in a variety of team sports [13].

Factors such as player age, competitive level, playing style and playing status are some examples of variables that can affect physical demands. In this regard, some studies reported significant differences between playing status, where starters presented higher values than non-starters in professional male and female soccer players [14–17]. However, those studies only examined variables such as training monotony, strain, acute:chronic workload ratios, while further analysis may provide additional key findings for coaches and performance staff. For instance, analysing accumulated load with and without match data may provide different interpretations and it is expected that starters and/or players with higher match participation present greater values of accumulated load [18]. Additionally, analyzing the relationship between total load, including match participation and wellness is warranted as wellness has previously been modulated by such factors [19]. The relationship between wellness and load is fundamental for coaches in order to understand the player’s state of fitness and freshness considering the total workload. Only in this way, is it possible to have an adequate and individualized load management [20]. Through a comprehensive and integrated monitoring strategy to assess changes in players (such as external load, internal load, wellness status, and readiness for training and match), can coaches implement appropriate training programs and recovery strategies to optimize player performance.

Some calculations such as training/match ratio (TMr) may also be extremely helpful to understand the physical demands induced by competitive match-play in starters and to further understand if compensatory training sessions are an adequate stimulus for non-starters [21, 22]. Furthermore, TMr may be fundamental to decreasing injury risk and optimizing performance [23]. The TMr are calculated by the division of the accumulated weekly load by the match load [22]. If the TMr provides a value equal to one, it suggests that the accumulated load of the week is equal to the match [22]. Usually, players in a standard micro-cycle should have a TMr of at least one, as exposure to match demands is the first prevention of the risk of injury, however, if excessively high, it also increases its risk of occurrence [24]. Even so, the oscillation between higher or lower values can vary greatly from context to context, from player to player and from metric to metric. League characteristics and game/training models can have an influence on what is accumulated training load during the week. In this way, there are teams that, due to their game model, end up accumulating more ACC/DEC than HSR and sprint [25], players who, due to their position, may be more exposed to certain types of efforts [26] and even individual characteristics that, like the moderators who influence how load is tolerated by players [27].

Due to scant recent studies, it would seem reasonable to compare starters and non-starters data [21]. For example, Stevens et al. [21] reported lower TMr values for non-starters compared to starters, specifically for high-speed running (HSR). However, Clemente et al. [22] analyzed the differences between varying micro-cycle schedules (3 versus 4 versus 5, but did not consider playing status). Nonetheless, the authors found that both TMr of HSR (HSRr) and TMr of sprint distance (SPDr) were higher in week in five sessions than weeks with three or four sessions. They concluded that training sessions were not adjusted according to weekly variations in match demands. Understanding individual TMr may be important to adjust training characteristics considering the needs of each player.

Therefore, the aims of this study were to: (i) compare accumulated load and wellness between starters and non-starters of a European professional soccer team; (ii) analyze the relationships between wellness and load measures and; (iii) compare TMr of external and internal load between starters and non-starters. The study hypothesis was that non-starters would present lower accumulated load and wellness values, and lower TMr when compared with starters [21]. Consequently, trivial-to-small correlations would be observed between load and wellness specifically for non-starters, considering that non-starters tend to present lower load during micro-cycles [21].

## Materials and methods

### Experimental approach to the problem

This was an observational study design. The players were monitored daily for wellness measures (sleep quality, muscle soreness, fatigue, stress and mood), internal and external load during 70 training sessions and 15 official matches over a 16-week period during the 2022/23 in-season period (July to November).

All micro-cycles were organized according to the following match, and thus all micro-cycles included only one match. In addition, when a player (non-starter) did not participate in MD, MD + 1 consisted of exercises to expose players to higher levels of HSR and sprinting distances and accelerations (ACC) and decelerations (DEC), while starters performed a recovery session. Therefore, to accomplish HSR and sprint distances, chasing opponents and goal-scoring exercises were performed, while small-sided games were performed to accomplish ACC and DEC targets. Some examples of scoring exercise included running to shoot on goal and then returning to defend the next player who will be shooting in the opposite goal, this way HSR and sprinting were accomplished. Ball possession exercises included two squares and three teams. The team that recovers the ball connects with the opposite square and the team that lost ball possession has to press immediately to regain the ball. Table 1 presents the number of training sessions and matches for each day of the micro-cycle.

**Table 1:** Number of training/match sessions of the 16-week period

Day of the week	MD + 1	MD-5	MD-4	MD-3	MD-2	MD-1	MD^a^
Number of sessions	13	6	9	13	15	16	16
Duration for starters (min)	24.2 ± 6.3	38.4 ± 2.6	44.8 ± 3.6	39.3 ± 1.0	35.2 ± 2.1	39.2 ± 5.6	80.2 ± 17.3
Duration for non-starters (min)	38.2 ± 6.5	39.0 ± 2.1	45.1 ± 3.5	40.8 ± 2.2	36.0 ± 2.0	26.6 ± 2.3	43.8 ± 16.3

*MD* Match day, *MD* + *1* One day after the match day), *MD-5* Five days before match daym *MD-4* Four days before match day, *MD-3* Three days before match day, *MD-2* Two days before match day, *MD-1* One day before match day

^a^for non-starters this was considered a training session

### Participants

Seventeen highly trained/national level professional male soccer players (age: 25 ± 2.8 years; body mass: 71 ± 6.6 kg; body height: 177.9 ± 6.7 cm; fat mass: 8.6 ± 1.0%; professional experience: 7.4 ± 2.7 years) participated in the current study. Players belonged to a European soccer team that played in the national first division. From the 17 players included, 7 were defenders, 5 were midfielders and 5 were attackers.

The eligibility criteria for participant inclusion were: (i) participating in 80% of the total number of training sessions (full session duration) [19] and; (ii) completed all wellness and training reports over the data collection period. In addition, players were considered starters if they started the match and completed at least 60-min in three consecutive matches while non-starters were the remaining players [14–17]. Thus, 10 players were considered starters (age: 25.1 ± 2.2 years; body mass: 73.4 ± 8.7 kg; body height: 180.7 ± 9.0 cm; fat mass: 8.7 ± 1.1%; professional experience: 7.3 ± 2.3 years), while seven were considered non-starters (age: 26.1 ± 4.6 years; body mass: 71.4 ± 7.2 kg; body height: 178.0 ± 6.3 cm; fat mass: 8.9 ± 1.5%; professional experience: 8.4 ± 4.2 years).

Prior to data collection, participants were fully informed of the study design and signed an informed consent. The study followed the ethical guidelines for human study as suggested by the Declaration of Helsinki (2013). Furthermore, the study was approved by the research Ethics Committee of the Polytechnic Institute of Santarém, Santarém, Portugal (Nº24-2022ESDRM).

### Wellness quantification

Following previous recommendations of overtraining monitoring [6], the wellness questionnaire of McLean et al. [7] was applied individually 30-min prior to each training/match session through a google form specifically designed. The questionnaire uses a scale of 1–5 arbitrary units (A.U.) and has five questions assessing fatigue, quality of sleep, muscle soreness, stress and mood (in which 5 is very fresh, very restful, very great, very relaxed and very positive mood, respectively, while 1 suggests always tired, insomnia, very sore, highly stressed and highly annoyed/irritable/down, respectively) [7]. All players were familiarized with the questionnaire during the previous full season.

### Internal load quantification

The CR-10 Borg’s scale [28] was employed to monitor players internal load (RPE) relative to the physiological and/or psychological effect of the work [1]. Using a valid and reliable practical tool is imperative for monitoring the training load imposed on athletes during every training session. Foster et al. [29] proposed a method based on RPE. This method, known as the session-RPE method, considers both the intensity and duration of a training session. Several studies confirmed the validity, reliability and internal consistency of the session-RPE method in soccer with players of varying levels [30, 31].

Following 20- to 30-min after each session, every player provided a perceived exertion value using a google form specifically designed by answering the following question: “how intense was the training session?”. The scale varied from 0 to 10 A.U., where each value rated as: 0 – nothing to all; 0.5 – extremely weak; 1 – very weak; 2 – weak; 3 – moderate; 4 – somewhat strong; 5 – strong; 7 – very strong; and 10 – extremely strong.

The score was used as a measure of internal intensity: RPE. In addition, the duration of the entire training session and/or match in minutes was multiplied by the RPE to generate the session-RPE (s-RPE) (A.U.) [29, 32]. All players were already familiarized with the questionnaire during the previous full season.

### External load quantification

Locomotor demands were measured using a 10 Hz GPS Vector S7 (Catapult Innovations, Melbourne, Australia). To avoid inter-unit bias, the same unit was used for each player throughout the analysis period [33]. The unit was placed on the upper back of the players, within a custom-made vest, 30-min prior to each session (training and match) and removed immediately post-session.

All data were transferred to a Microsoft Excel spreadsheet from Catapult Openfield software (Version 1.21.1). The data were grouped by training session, although only actual training time was considered for analysis, that is, data from the warm-up onwards, with transition time between exercises and rest times removed.

The GPS device has previously been validated for accuracy and reliability regarding sprint acceleration profiles [34] and in measures such as distance, speed and average acceleration where the coefficient of variation ranged from 0.1 to 3.9% (reliability for running distance measures and average acceleration) [35]. The following metrics were used for analysis: (i) high-speed running (HSR, 20–25 km/h) and sprinting (SPD, > 25 km/h) based on Houtmeyers et al. [36], number of accelerations (ACC, > 2 m/s^2^) and number of decelerations (DEC, < 2 m/s^2^) based on Gonçalves et al. [37].

### Accumulated wellness/load and training/match ratio

Accumulated wellness/load consisted of the sum of each measure during all training sessions of the micro-cycle and was calculated per player, thus providing the weekly load for each measure (MD included).

Moreover, accumulated load was also calculated without MD data in order to calculated training/match ratio (TMr) for all internal and external measures. Ratios were then calculated by dividing accumulated load (without MD data) by MD data (TMr = weekly load/match demands) [22]. This calculation was made for each player and then, average values were used for each group (starters and non-starters). Consequently, the following measures were obtained: RPEr (rating of perceived exertion ratio); s-RPEr (session rating of perceived exertion ratio); HSRr (high-speed running ratio); SPDr (sprinting distance ratio); ACCr (accelerations ratio) and; DECr (decelerations ratio). The same ratio was calculated for session duration (Dr).

### Statistical analysis

Descriptive statistics are presented as mean ± standard deviation. Normality and homogeneity of the different variables was tested using the Shapiro–wilk and Levene tests, respectively. Only wellness variables did not present normal distribution (*p* < 0.05). Thus, Mann–Whitney U test was used for wellness variables while independent T-test was used for the remaining variables. Significant results were considered at *p* < 0.05, while the Hedges effect-size was performed to determine the effect magnitude through the difference of two means divided by the standard deviation from the data and the following criteria were used: < 0.2 = trivial, 0.2 to 0.6 = small effect, 0.6 to 1.2 = moderate effect, 1.2 to 2.0 = large effect, and > 2.0 = very large [38].

Finally, the relationship between wellness and load variables were explored using the Spearman’s Rho correlation coefficient. The magnitude of correlations were classified as trivial (0.00 to 0.09), small (0.10 to 0.29), moderate (0.30 to 0.49), large (0.50 to 0.69), very large (0.70 to 0.89), and nearly perfect (> 0.90) [39].

All statistical procedures were executed in the IBM SPSS Statistics for Windows version 23.0 (IBM Corp, Armonk, NY:USA).

## Results

Table 2 presents the accumulated training demands (MD data included) for all variables. Non-starters presented significantly higher values for fatigue and lower significant values for duration and s-RPE when compared with starters. There were no other significant differences.

**Table 2:** Descriptive statistics (mean ± standard deviation, and confidence interval, CI, 95%) for accumulated wellness and load demands

Variables	Starters	Non-starters	*p*-value	Effect size
Quality of sleep (A.U.)	26.2 ± 2.4 (24.5–27.9)	26.9 ± 3.0 (24.1–29.7)	0.536	-
Fatigue (A.U.)	22.8 ± 3.0 (20.6–25.0)	26.3 ± 2.7 (23.8–28.8)	0.019*	0.24
Muscle Soreness (A.U.)	22.4 ± 12.8 (19.9–25.0)	25.5 ± 3.0 (22.7–28.3)	0.070	-
Stress (A.U.)	24.4 ± 3.5 (22.4–27.4)	25.2 ± 3.8 (21.7–28.7)	0.740	-
Mood (A.U.)	24.9 ± 3.1 (22.7–27.2)	25.1 ± 4.0 (21.4–28.0)	> 0.999	-
RPE (A.U.)	36.4 ± 2.0 (34.9–37.8)	35.7 ± 3.0 (32.9–38.5)	0.594	-
Duration (min)	291.3 ± 11.9 (282.8–299.9)	269.5 ± 16.4 (254.4–284.7)	0.006*	1.81
s-RPE (A.U.)	1837.3 ± 105.6 (1761.8–1912.9)	1552.4 ± 170.5 (1394.7–1710.2)	0.001*	2.69
High speed running (m)	1107.9 ± 208.9 (208.9–1257.4)	1132.4 ± 86.3 (1052.6–1212.2)	0.745	-
Sprinting (m)	278.9 ± 87.2 (216.6–341.3)	320.0 ± 54.6 (269.5–370.5)	0.289	-
Accelerations (nr)	291.7 ± 37.0 (265.2–318.2)	306.4 ± 21.1 (286.9–325.9)	0.359	-
Decelerations (nr)	268.0 ± 26.9 (248.0–289.3)	283.3 ± 28.3 (257.1–309.5)	0.315	-

*RPE* Rate of perceived exertion using the CR-10 Borg’s scale, *s-RPE* Multiplication of session time by the RPE score, *A.U* Arbitrary units, *m* Meters, *min* Minutes, * denotes significant difference between starters versus non-starters (*p* < 0.05)

Table 3 presents the correlation coefficients among wellness and the physical demands using the accumulated micro-cycle (including the MD) values for starters. Significant positive and nearly perfect correlations were found between fatigue and muscle soreness, muscle soreness and mood, and stress and mood. In addition, significant positive very large correlations were found between fatigue and stress, muscle soreness and stress, fatigue and mood, and ACC and DEC. Finally, there was a significant large correlation between HSR and ACC and HSR and DEC.

**Table 3:** Correlation coefficient (r) for wellness and load demands in starters

Variables	Fatigue	Muscle Soreness	Stress	Mood	RPE	Time of session	s-RPE	HSR	Sprinting	ACC	DEC
Quality of sleep	0.527	0.527	0.503	0.479	-0.333	-0.030	-0.588	0.079	0.079	0.442	0.115
Fatigue		**0.915** ***p* < 0.001**	**0.745** ***p*** **= 0.013**	**0.782** ***p*** **= 0.008**	-0.030	-0.139	-0.188	0.236	0.236	0.467	0.491
Muscle Soreness			**0.806** ***p*** **= 0.005**	**0.915** ***p* < 0.001**	0.030	-0.370	-0.345	0.358	0.224	0.370	0.382
Stress				**0.903** ***p* < 0.001**	-0.224	0.030	-0.115	0.030	-0.224	0.067	0.127
Mood					-0.055	-0.188	-0.333	0.103	-0.006	0.067	0.127
RPE						-467	0.188	0.261	0.479	0.261	0.406
Time of session							0.527	-0.442	-0.200	-0.333	-0.236
s-RPE								0.115	0.103	-0.103	0.224
HSR									0.612	**0.648** ***p*** **= 0.043**	**0.685** ***p*** **= 0.029**
Sprinting										0.430	0.430
ACC											**0.891** ***p*** **= 0.001**

*RPE* Rate of perceived exertion using the CR-10 Borg’s scale, *s-RPE* Multiplication of session time by the RPE score, *HSR* High speed running (20–25 km/h), *ACC* Acceleration, *DEC* Deceleration, Bold denotes significant correlations

Table 4 presents the correlation coefficients among wellness and physical demands using the accumulated micro-cycle (including the MD) values for non-starters. Significant positive and nearly perfect correlations were found between: stress and mood. Moreover, significant positive very large correlations were found between fatigue and muscle soreness, quality of sleep and RPE. Finally, significant negative very large correlations were found between stress and DEC, and mood and DEC.

**Table 4:** Correlation coefficient (r) for wellness and load demands in non-starters

Variables	Fatigue	Muscle Soreness	Stress	Mood	RPE	Time of session	s-RPE			HSR	Sprinting	ACC	DEC
Quality of sleep	0.321	0.321	0.179	0.286	**0.893** ***p*** **= 0.007**	0.143		0.714		0.214	-0.286	-0.036	-0.357
Fatigue		**0.786** ***p*** **= 0.036**	0.321	0.393	0.107	0.464		0.250		-0.393	-0.250	-0.536	-0.429
Muscle Soreness			0.714	0.750	0.179	0.679			0.536	-0.071	< 0.001	-0.536	-0.643
Stress				**0.964** ***p*** **< 0.001**	-0.071	0.179			0.250	0.286	0.071	-0.750	**-0.786** ***p*** **= 0.036**
Mood					0.071	0.143		0.286		0.214	0.036	-0.786	**-0.857** ***p*** **= 0.014**
RPE						0.214		0.750		0.250	-0.071	0.286	-0.107
Time of session								0.714		-0.241	-0.036	0.214	-0.143
s-RPE										0.071	-0.214	0.250	-0.357
HSR											0.714	< 0.001	0.214
Sprinting												0.036	0.464
ACC													0.679

*RPE* Rate of perceived exertion using the CR-10 Borg’s scale, *s-RPE* Multiplication of time of session by the score of RPE, *HSR* High speed running (20–25 km/h), *ACC* Acceleration, *DEC* Deceleration, Bold denotes significant correlations

**Fig. 1:**
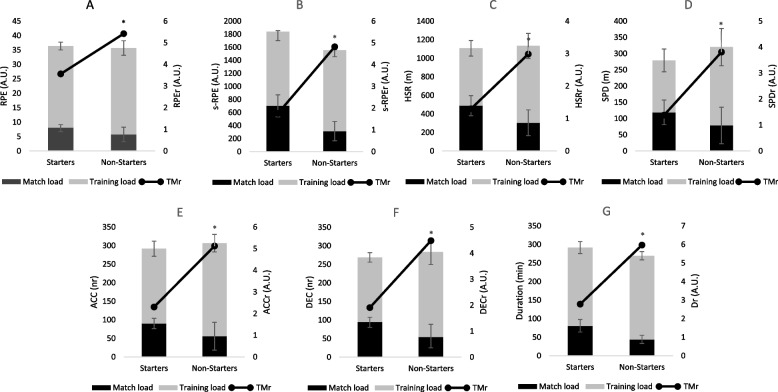
Accumulative weekly match load, training load, and training/match ratios for RPE, s-RPE, HSR, SPD, ACC, DEC and Duration. RPEr: rating of perceived exertion ratio; s-RPEr: session rating of perceived exertion ratio; HSRr: high-speed running ratio; SPDr: sprinting distance ratio; ACCr: accelerations ratio; DECr: decelerations ratio; Dr: duration ratio. * denotes significant difference for starters (*p* < 0.05)

Figure 1 shows that non-starters presented higher values in all TMr than non-starters, namely, RPEr (*p* = 0.001; g = 1.96), s-RPEr (*p* = 0.002; g = 1.77), HSRr (*p* = 0.001; g = 2.02), SPDr (*p* = 0.002; g = 4.23), ACCr (*p* = 0.001; g = 2.72), DECr (*p* < 0.001; g = 3.44), Dr (*p* = 0.003; g = 2.27).

## Discussion

The aims of this study were to: (i) compare accumulated load and wellness between starters and non-starters of a European professional soccer team; (ii) analyze the relationship between wellness and load measures and; (iii) compare TMr of external and internal load between starters and non-starters. The main results of the study reported that non-starters presented significantly higher values for fatigue and significantly lower values for duration and s-RPE when compared with starters. In addition, starters showed positive correlations between fatigue and muscle soreness, muscle soreness and mood, stress and mood, fatigue and stress, muscle soreness and stress, fatigue and mood, ACC and DEC, HSR and ACC, and HSR and DEC. While non-starters reported correlations between stress and mood, fatigue and muscle soreness, quality of sleep and RPE, and negative correlations between stress and DEC, and mood and DEC.

There is a lack of evidence comparing differences between the varying status of players (starter versus non-starter) of load and wellness measures, thus this may partly be explained by the specification of MD + 1 training. In these training sessions, non-starters performed compensatory training that focussed on physical demands to ensure these players were provided with a stimulus similar to that of starters (from the previous match). However, the characteristics of these training tasks for non-starters, sometimes with more analytical exercises, focusing on fitness development and less specific dynamics of the game [40], resulted in higher values for duration and s-RPE on MD + 1 (for instance, starters presented 24 min and 101.8 A.U. while non-starters 38 min and 230.6 A.U. of duration and s-RPE, respectively). In this regard, some authors [41] have reported that wellness measures have sometimes revealed an element of subjectivity when associated with load monitoring variables measured through more conventional objective markers. Furthermore, it has also been argued that despite the growing interest and use of wellness measures, there is still a lack of a general frame referenced in the current literature [42].

Nonetheless, the other notable study result was that TMr comparisons reported higher values for non-starters in all variables despite lower match and training durations when compared with starters. Once again, it may be speculated that the compensatory training session (MD + 1) as a strategy to resolve differential weekly training load between starting and non-starting players’ may partly explain these results, specifically highlighting the effort and commitment during match minutes. Regarding the first aim, the present study results are not in accordance with previous research, that showed higher fatigue, stress and muscle soreness levels for starters compared with non-starters over a 40-week period [17]. Such findings were also corroborated by Nobari et al. [18] that found higher weekly acute s-RPE, fatigue, and stress occurred in starters when compared with non-starters at the end of the competitive season, albeit in elite young soccer players [43]. Furthermore, it is relevant to note that despite the non-significant differences in external load measures, starters showed lower values in comparison to non-starters thus contradicting previous literature that attributed differences to match playing time [44, 45]. The findings of the existing literature do not fully explain the present results as accumulated duration was higher for starters. Thus, the external load of the study players seems to be adjusted appropriately for starters and non-starters.

Considering the second aim of the study, the most relevant associations occurred between load and wellness measures. In this regard, starters only showed an association within wellness measures (e.g., fatigue and muscle soreness) and within external load measures (e.g., sprint and acceleration), respectively. However, non-starters highlighted associations between the quality of sleep and RPE, stress and DEC, and mood and DEC. A study examining youth soccer players found higher external and internal intensity to be associated with improved sleep (quality and quantity), and feeling rested, although did not consider playing status [46] that contrasts the present results. Contrastingly, a different study in youth soccer players found that high-intensity training had no impact on the following night’s sleep quality [47]. While a further study conducted with professional soccer players also reported that sleep quality was not affected by higher intensity sessions (MD included) [48]. Again, these previous studies did not consider the starting status of players, starters and non-starters. Thus, more research is warranted to investigate these specific groups. Nonetheless, the relationship between perceived exertion and a higher number of DEC seems to decrease wellness, namely, stress and mood while sleep is improved for non-starters.

Furthermore, relevant findings of the current study were related to aim (iii), where non-starters revealed higher TMr in all variables regardless of the lower match and training durations when compared with starters. Such findings are in contrast with some studies that attributed higher match-play minutes as a possible explanation for such differences. However, those studies found higher values of HSR, sprint distance, number of ACC and number of repeated sprints for starters [44, 45, 49]. This may suggest that coaches and performance staff adjusted training sessions for non-starters regardless of match participation. Considering the comparison of starters and non-starters, it seems that only Stevens et al. [21] conducted such analysis utilizing TMr. The authors found lower TMr values for non-starters when compared to starters with significant differences for HSR, while the remaining running metrics and accelerometry variables were also higher for starters, thus contradicting the current study findings.

Additionally, the calculation of TMr using both RPE and s-RPE was, to our knowledge, the first study to report such a method. Nonetheless, the external TMr values found in the present study for starters ranged from 1.3 to 2.3 A.U, while non-starters presented a range between 2.9 and 5.1 A.U, that is similar to those previously reported by Clemente et al. [22] who found a range of ~ 1 to 4 A.U for Portuguese soccer players. Although, these data were higher than those reported by Stevens et al. [21] for HSR (0.22) and sprint distance (0.03) in Dutch soccer players. However, such ratios are dependent on the number of training sessions per week. Therefore, possible explanations for the different values may be associated with different training strategies that included additional conditioning exercises and/or supplementary sessions that may have been conducted as previously suggested [22]. Nevertheless, in the present study it was found that non-starters had higher TMr values for ACC and DEC which seems to suggest that additional training for non-starters (performed on MD + 1) consisted of small-sided games, as this game format increases the number of ACC/DEC and decreases HSR and sprint distances covered [50, 51].

Notwithstanding, the present study had some limitations. The main limitation is the small sample size that derived from only one team, and across a restricted time period of 16-weeks. Furthermore, as reported in Table 1, different training days per week occurred that may have influenced TMr values. Even so, average values were employed and produced relevant information for coaches and performance staff. Moreover, playing position differences were not considered for analysis and for some positions, such as wide players (attacking and defending), perform higher accumulated values, TMr and the perceived various wellness status, as these positions produce greater running effort than other positions [52]. Consequently, future studies should attempt to analyze larger sample sizes, include different training frequencies [22] and analyze regular weeks with one match versus congested weeks with more than one match as previously suggested [21]. Additionally, the generalization of these results to other teams/countries, competitive standards and ages is not recommended and thus further replication studies are required. For instance, recently no external load difference was observed between an under-18 and a first team [53], although the analysis of TMr would provide more insights for coaches. Finally, a detailed description of training drills in future research may facilitate a more precise practical implementation.

Although these results may be dependent on the analyzed team, this study showed that it is possible to plan higher load for non-starters during the in-season without negatively impacting training for starters. From a load perspective, non-starters need to be physically prepared to replace or substitute a starter. Moreover, TMr analysis facilitates the interpretation and contextualization of data, and consequently allows the training prescription to be planned accordingly to achieve the appropriate load and also allows coaches and performance staff to communicate effectively with each other and players as previously suggested [21]. Finally, the individuality and how athletes recover varies from player to player. Some athletes perceive a high load and recover well, while other athletes recover slower, hence the moderators explain that absolute and/or equal load can be tolerated differently. Thus, this supports the notion to interpretate results with caution.

## Conclusions

In summary, it was observed that non-starters presented significantly higher values for fatigue (suggesting less fatigue) and lower significant values for duration and s-RPE than starters. Specifically, non-starters reported higher TMr in all variables despite lower match and training durations when compared with starters suggesting that physical load was adjusted appropriately.

Furthermore, non-starters showed positive relationships between stress and mood, fatigue and muscle soreness, and quality of sleep and RPE. Moreover, non-starters reported negative relationships between stress and DEC and mood and DEC. These findings seemed to suggest that perceived exertion and a higher number of DEC may contribute to decreased wellness, namely, quality of sleep and stress and mood for non-starters.The current study also provides practical information regarding the micro-cycle periodization, and suggests that the type of exercises used during the compensatory training sessions were appropriate for this specific group. Finally, although this study provides average values, it is not recommended to apply these findings to other teams and coaches from similar contexts.

## Data Availability

The data presented in this study are available on request from the corresponding author.

## Abbreviations

TMr: Training/match ratio
MD: Match day
MD + 1: One day after the match day)
MD-5: Five days before match day
MD-4: Four days before match day
MD-3: Three days before match day
MD-2: Two days before match day
MD-1: One day before match day
RPE: Rating of perceived exertion
s-RPE: Session rating of perceived exertion
HSR: High-speed running
ACC: Acceleration
DEC: Deceleration
GPS: Global positioning system
A.U.: Arbitrary units
RPEr: Rating of perceived exertion ratio
s-RPEr: Session rating of perceived exertion ratio
HSRr: High-speed running ratio
SPDr: Sprinting distance ratio
ACCr: Accelerations ratio
DECr: Decelerations ratio
Dr: Duration ratio
